# Aging and Options to Halt Declining Immunity to Virus Infections

**DOI:** 10.3389/fimmu.2021.681449

**Published:** 2021-05-12

**Authors:** Miguel Ángel Palacios-Pedrero, Albert D. M. E. Osterhaus, Tanja Becker, Husni Elbahesh, Guus F. Rimmelzwaan, Giulietta Saletti

**Affiliations:** Research Center for Emerging Infections and Zoonoses, University of Veterinary Medicine Hannover, Hannover, Germany

**Keywords:** aging, cell mediated immunity, virus infections, vaccine, immunosenescence

## Abstract

Immunosenescence is a process associated with aging that leads to dysregulation of cells of innate and adaptive immunity, which may become dysfunctional. Consequently, older adults show increased severity of viral and bacterial infections and impaired responses to vaccinations. A better understanding of the process of immunosenescence will aid the development of novel strategies to boost the immune system in older adults. In this review, we focus on major alterations of the immune system triggered by aging, and address the effect of chronic viral infections, effectiveness of vaccination of older adults and strategies to improve immune function in this vulnerable age group.

## Introduction

Life expectancy is currently higher than ever and by 2050 the number of individuals over 65 years of age is estimated to be more than 1.6 billion worldwide (https://www.who.int/news-room/fact-sheets/detail/ageing-and-health). Aging is associated with progressive changes involving all organ systems including the immune system, collectively affecting the ability to mount protective immune responses to various infectious pathogens and to vaccination (1–9). Therefore, age-related diseases and conditions are of major public health concern and should prompt the development of more effective therapies and vaccines to prevent or mitigate the impact of infectious diseases that are a major cause of morbidity and mortality in older adults (10–13). For instance, the recent outbreak of COVID-19 caused by SARS CoV-2 has highlighted the increased severity of this virus infection in older adults, resulting in disproportionally high morbidity and mortality rates (14–19).

The term “immunosenescence” refers to alterations and dysregulation of the immune system that take place during aging. It is a multifaceted, biological process causing a progressive malfunctioning of cells of innate and adaptive immunity. Changes in both composition and function of immune cells characterized by up- or down-regulation of surface markers, defects in cell signaling and alterations of cell populations are hallmarks of immunosenescence. On the one hand, genetic background, microbiome, diet, co-morbidities and/or environmental factors are thought to play a role in the aging process (20, 21). Immunosenescence has also been reported in some animal species including non-human primates and dogs (22–24). On the other hand, it should be considered that: (i) many older adults remain healthy until advanced age (>90 years of age); (ii) in some cases “dysfunctional” immune cells can function properly when stimulated adequately, e.g., with the use of vaccines designed for use in older people; (iii) altered response not always translates into being harmful (25, 26).

The innate immune system plays a key role as the first line of defense against pathogens and promotes the adaptive immune system to generate long-lasting protective immunity. In case of vaccination, the functionality of innate immune cells at the site of administration (e.g., macrophages, dendritic cells, neutrophils) is of importance and largely dictates the outcome of vaccination. In older adults, it has been reported that antigen processing and presentation capacities are reduced and neutrophil cytokine production is altered, leading to dysfunctional chemotaxis and activation of other immune cells (27–33). Upon infection or vaccination, a small number of naive CD4^+^ and CD8^+^ T cells recognize a cognate peptide/MHC complex presented by antigen presenting cells, which undergo activation, proliferation and differentiation into effector cells. After the contraction phase, the majority of the effector cells (90-95%) die by apoptosis and only a small fraction of the CD4^+^ and CD8^+^ T cell effector population differentiate into memory cells (34–36). These cells are responsible for maintaining long-term immunity and protective recall responses. Older individuals experience a decrease in the number of naive T cells due to reduced hematopoiesis and thymus involution, which impairs the response to novel antigens, including vaccine antigens (37–41). Nonetheless, naive T cells from individuals older than 70 years still exhibit highly diverse T cell receptor (TCR) specificities, albeit reduced compared to those of younger adults (38, 40, 42). Furthermore, immunosenescent T cells are characterized by an impaired proliferation rate, resistance to apoptosis, downregulation of co-stimulatory surface molecules (e.g., CD27 and CD28), telomere shortening and expression of KLRG-1 and CD57 (43–55). Under normal circumstances, the fine-controlled balance between pro-inflammatory and anti-inflammatory cytokines maintains the physiologic function of inflammation. In older people, dysregulation of cytokine production with a progressive tendency toward a pro-inflammatory phenotype (e.g., IL-6, IL-15, IFN-γ), is believed to play a key role in the inability to control systemic inflammation. This chronic low-grade of uncontrolled inflammation is defined as “inflammaging” (56–64). Recent studies have shown that persistence of chronic inflammation is associated with various environmental and metabolic factors (e.g., diet, nutrition and gut microbiota) (65–67). Pro-inflammatory cytokines are also secreted in response to oxidative stress, DNA damage and autophagy [reviewed elsewhere (68, 69)]. The processes of immunosenescence and inflammaging are intertwined with each other. On the one hand, senescent cells, which accumulate during aging, are characterized by a pro-inflammatory cytokine secretion pattern, leading to inflammaging. On the other hand, an increase of such inflammatory mediators drives altered adaptive immune responses, contributing to immunosenescence. Both processes are therefore involved in a vicious cycle that impairs functioning of the immune system in older adults (70–72). In addition, chronic infections (e.g., caused by cytomegalovirus) contribute to the enhancement of both, immunosenescence and inflammaging. Such pathogens trigger a low but continuous inflammatory response in the host, as well as a clonal expansion of differentiated/memory T cells at the cost of naive T cell compartment, contributing to an impaired immune response to novel pathogens (73–78).

The second arm of the adaptive immune response is mediated by humoral immunity. Humoral immunity is a key correlate of protection against infectious pathogens and the induction of specific antibodies and the development of specific B cell memory are pivotal to provide protection against reinfection and contribute to the success of vaccination. Aging has a profound impact on diversity of the B cell repertoire. Impaired somatic hypermutation and isotype switching, together with a reduction in the number of plasma cells, affect the magnitude and quality of the antibody response induced by infection or vaccination (41, 79–82).

Aging also has a major impact on the severity of viral and bacterial infections, resulting in a more severe course and often fatal outcome in the older population. Furthermore, effectiveness and longevity of vaccination, the main preventive measure against infections, are gradually reduced in the aging population. Certain viruses can establish a low level of viral replication and cause persistent and often latent infections. In immunosenescent adults, these viruses can reactivate and cause a severe infection that can lead to recurrent disease of increasing severity. Varicella zoster virus (VZV) infection is a notable example of this. VZV, an α-herpesvirus, usually causes infections during childhood (chickenpox) and then becomes persistent and latent. Virus reactivation leads to shingles, also known as herpes zoster (HZ), and possible further serious complications (83–88). Another example is the human cytomegalovirus (CMV), which is highly prevalent in adults worldwide. CMV, a β-herpesvirus, also establishes life-long latent infection and is generally considered to promote T cell immunosenescence (89–91). CMV infection leads to continuous or chronic antigen presentation, resulting in increased numbers of highly differentiated T cells with a reduced TCR repertoire (62, 92–96).

In addition, other (respiratory) viruses, such as respiratory syncytial virus (RSV) and influenza viruses (IVs), cause more severe respiratory disease with complications and mortality in older adults, especially in association of co-morbidities (97–102). Although effective influenza vaccines are available, their effectiveness is not optimal in older people. Attempts to develop more immunogenic vaccine formulations for this age category have resulted in somewhat more effective influenza vaccines (103–106). In contrast, a registered RSV vaccine is still not available, although several candidate RSV vaccines are in various stages of development (107–110).

In this review, we discuss the current understanding of age-related changes affecting immune cells and how that influences protective immune response to virus infection and vaccination. Moreover, we will give an insight into strategies that are currently used, or under development, to improve immune cell functions and vaccine efficacy in older adults.

## Age-Associated Immune Cell Dysfunctions

The immune system is complex and involves the interplay of many different cell types that collectively afford protection against infectious pathogens. Aging negatively influences the function of immune cells resulting in increased severity of infections and impaired responses upon vaccination. Although there is an increasing interest in the development of therapeutic and preventive strategies targeting older adults, many molecular processes leading to immunosenescence are still not fully understood. Additionally, a large body of knowledge has been obtained from mouse models; however, it is not clear to what extent this model faithfully represents the mechanisms controlling these processes in humans. Furthermore, most studies have used peripheral blood mononuclear cells (PBMCs) because of ease of accessibility, but these do not account for potential changes of cells residing in tissues. Consequently, more research is required to obtain a better understanding of immunosenescence in aging humans, which will guide the design of improved vaccines tailored for use in the aging population.

Some of the factors that are known to play a role in immunosenescence during aging include genomic instability, telomere shortening, epigenetic modifications, changes of intracellular signal pathways responsible for cell communication and dysfunction of mitochondria [reviewed elsewhere (8, 111)].

**Figure 1:**
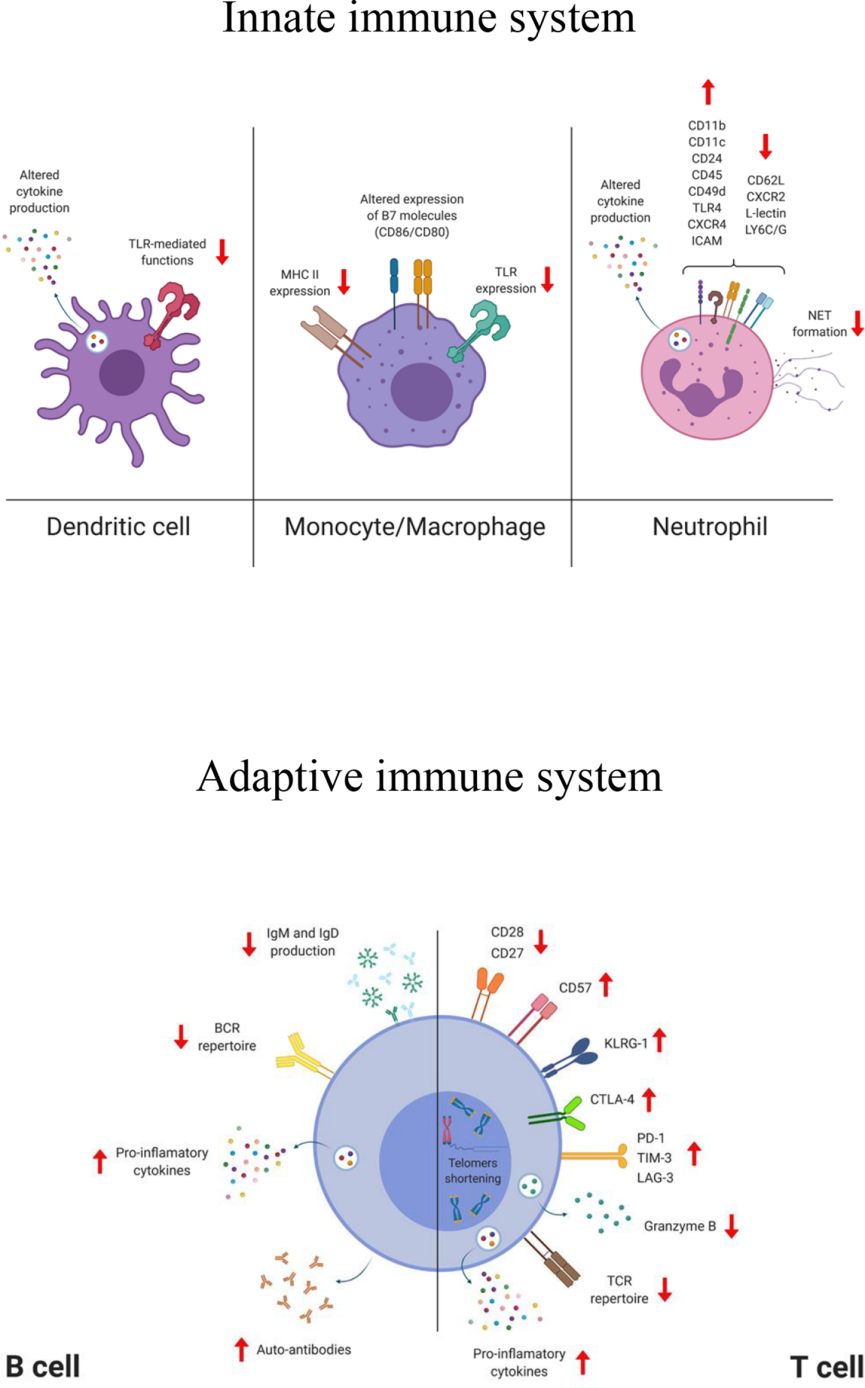
Simplified representation of the phenotypical and functional changes affecting cells of the innate and adaptive compartment during aging. TLR, toll like receptor; MHC, major histocompatibility complex; CXCR, C-X-C chemokine receptor; ICAM, intercellular adhesion molecule; LY6C/G, lymphocyte antigen 6 complex (locus C/G); NET, neutrophil extracellular trap; BCR, B cell receptor; TCR, T cell receptor; KLRG-1, killer-cell lectin like receptor G1; CTLA-4, cytotoxic T-lymphocyte antigen 4; PD-1, programmed cell death protein 1; TIM-3, T cell immunoglobulin and mucin domain-containing protein 3; LAG-3, lymphocyte-activation gene 3. The figure was created with BioRender.com.

Here we will focus on age-related changes of immune cells of both innate and adaptive immunity, along with those induced after infection or vaccination (**Figure 1**).

### Innate Immune System

*Dendritic cells* (DCs) are critical for the induction of protective immune responses against pathogens and represent the link between innate and adaptive immunity. DCs are professional antigen-presenting cells that provide activation signals to T cells and are crucial for mounting a robust humoral response (112–114). DCs can be activated by various stimuli including microbes, apoptotic cells, and inflammatory cytokines. They sense pathogens at the port of entry through pattern recognition receptors (PRRs) (e.g., Toll-like receptors, C-type lectin receptors, intracytoplasmic NOD-like receptors), and then migrate to the lymphoid organs where they prime naive T cells and regulate B cell responses. DCs are comprised of two major subsets of either myeloid (mDCs; conventional/classical DC, cDC) or lymphoid origin (plasmocytoid; pDCs). mDCs regulate pro-inflammatory responses (e.g., *via* T-helper 1 and cytotoxic T lymphocyte responses) upon bacterial and viral infections, and pDC produce type I interferon, that can directly antagonize viral replication (115–118). It has been shown that magnitude, functionality and signaling of the pDCs are reduced in older people, and that has been correlated with the increased occurrence of severe influenza virus infections in this age group (30, 119, 120). Yet, impaired T cell proliferation and IFN-γ secretion have been observed upon stimulation of aged pDCs with influenza virus (121). Transcriptional analyses have demonstrated that PBMCs from people over 65 years of age display a delayed and atypical response after stimulation *via* some Toll-like receptors (TLR4 and TLR7/8) compared to those from younger individuals (<40 years old), which has a profound effect on the production of effector molecules involved in T cell activation and proliferation, such as IFN-γ and TNF-α (122). Following TLR7/9 stimulation, pDCs of older subjects produce less type I and III interferons and display a reduced antigen presentation that impairs T cell activation (121). An increased level of IL-6 and TNF-α secreted by “old” mDCs in response to LPS, ssRNA, and self-DNA, has been associated to alteration in signaling pathways that lead to PI3K, NF-κB, or type I IFN responses (123, 124). A decreased expression and/or function of some TLRs in DCs and monocytes in older adults individuals has also been reported (125–127). Moreover, epigenetic changes (e.g., methylation or phosphorylation) induce up-regulation of immune checkpoint molecules on DCs that reduce the ability to migrate to the secondary lymphoid, leading to an impaired T cell activation (114, 128–132). The development of a robust and protective antibody response with generation of high-affinity antibodies is mediated by the ability of follicular DCs (fDCs) to capture and retain, for extended periods of time, native antigen in the B cell follicles within the germinal center. Studies have documented that age-related defects in the germinal center formation and reaction (e.g., retention of antigen complexes and reduced expression of the FcγRII on fDCs), have a dramatic impact on generation of humoral immunity (133).

*Monocytes* and tissue-resident *macrophages*, which can be monocyte-derived or embryo-derived, are phagocytic cells, primarily involved in the innate immune response against infectious pathogens and also involved in the maintenance of tissue homeostasis (134). Age-related defects in the macrophage/monocyte lineage function are predominantly mediated by the dysregulation of cellular signaling, which affects antigen presentation and response to inflammatory stimuli (135–137). Macrophages are usually divided in two subsets M1 (pro-inflammatory) and M2 (anti-inflammatory/immune regulatory) and aging alters their polarization and function (137–139). Due to the difficulty in obtaining and studying tissue-resident macrophages and their highly diverse tissue-dependent phenotypes, it is still not completely understood which macrophages/tissue are more affected by aging (140–142). Macrophages may also contribute to the chronic inflammatory state in older adults and lead to the progression of age-associated diseases. Although no differences have been found in the number of peripheral blood monocytes in older adults compared to younger subjects, there is an age-dependent upward shift in the proportion of monocyte subsets with an inflammatory profile. For example, ‘non-classical’ CD14^low^CD16^+^ monocytes specifically increase with age and display reduced HLA-DR and CX(3)CR1 surface expression (143). Upon TLR stimulation, monocytes display age-dependent differences, both at transcriptional and functional levels, resulting in a differential expression of surface molecules and cytokine production (144). These age-related alterations have been mainly associated to impaired monocyte TLR responses, although few studies show a rather enhanced inflammatory response (125, 144–146). For example, a positive correlation was found between surface expression of B7 co-stimulatory molecules before influenza vaccination and the vaccine induced antibody response, but interestingly, this correlation was weaker for older vaccinees (147–149). Furthermore, expression of MHC class II molecules was lower in aged human monocytes and mouse macrophages (150–153). Moreover, aged human monocytes have shortened telomeres, reduced phagocytic capacity and high level of intracellular TNF-α (154).

*Neutrophils* are phagocytic cells that migrate from blood to infected tissues and rapidly respond to invading pathogens by activating adhesion, chemotaxis, phagocytosis as well as release of oxidants, proteases and other molecules (155–158). However, an excessive accumulation of neutrophils and hyper-responsiveness can be detrimental and cause tissue injury, as recently documented in subjects with lethal COVID-19 (159, 160). Although no difference has been found in the number of neutrophils between young and older adults, aged neutrophils exhibit a dysfunctional phagocytic and chemotactic capacity (28, 161, 162). In aged mice, these dysfunctional cells expand and accumulate in lymph nodes due to impaired apoptosis (163, 164). Phenotypically, up-regulation of CD45, TLR4, CD24, CXCR4, CD11b, CD11c, CD49d, ICAM and down-regulation of CXCR2, CD62L, L-lectin, LY6C/G have been documented in aged human neutrophils (156, 165). In addition, altered cytokines secretion profiles, generation of reactive oxygen species (ROS) and associated microbial killing have been reported, although these findings might largely be stimulus-dependent (28, 166, 167). Age dependent impaired formation of neutrophil extracellular traps (NETs), structures able to capture and immobilize pathogens, may explain why older adults are more susceptible to invasive bacterial infections (29, 168, 169).

*Natural killer (NK) cells* are cytotoxic innate cells that eliminate infected, transformed and senescent cells (170–175). In addition, they also exert immunoregulatory activities by secreting cytokines and chemokines, which can activate and modulate the adaptive immune responses (176–178). Like other cells, NK cells are subject to age-related changes in, for instance, number, function, phenotype and redistribution (179, 180). As an example, there is an increased number of highly differentiated mature CD56^dim^ cells accompanied with a decline of immature CD56^bright^ subset as well as reduced NK cell activity, which may lead to impaired immune regulation (181–184).

### Adaptive Immune System

Profound age-related changes in the immune system are observed in cells of adaptive immunity. In this section, we will describe major alterations affecting aged T and B lymphocytes.

#### T Lymphocytes

T cells recognize antigens derived from pathogens or tumors *via* their T cell receptor and develop antigen-specific memory responses or tolerance (185). Upon activation, naive T cells proliferate, differentiate and generate effector T cells that can help to kill infected cells and/or to activate other cells (e.g., macrophages), and B cells. T cell precursors are generated within the bone marrow whereas their maturation and selection take place in the thymus. The thymus undergoes an involution process starting after puberty that gradually induces its atrophy (186, 187). Epithelial cell structure and cell turnover are altered and there is a shift toward adipose tissue that results into a reduced thymopoiesis, as well as altered transcription factors and cytokine production (40, 188). As a consequence, the output of newly generated naive T cells (CCR7^+^, CD62L^+^, CD45RO^-^, CD45RA^+^, CD27^+^, CD28^+^), and therefore the possibility to respond to novel pathogens, is impaired (38, 189, 190). Despite the compensatory mechanism that promotes homeostatic proliferation of existing naive cells, their frequency is reduced in the periphery and in lymphoid organs (38, 42, 191–195). Coinciding with the loss of naive T cells, highly differentiated effector/memory T cells accumulate during aging, especially CD8^+^ T cells, many of which are dysfunctional. In particular, altered cytokine production profiles, reduced TCR clonal diversity and more self-reactive T cells have been observed (196–200) together with a general decline of the proliferative capacity in response to TCR stimulation, with CD4^+^ memory T cells more prone to this loss of function (201, 202). An increased frequency of a subset of memory CD8^+^ T cells with a naive phenotype (TMNP) that secretes effector molecules (e.g., IFN-γ, Granzyme B) in response to chronic viral stimulation has been associated with aging. The frequency of these cells positively correlates with the severity of West Nile and influenza virus infections (203). Furthermore, regulatory T cells (Tregs) with up-regulated check point molecules, such as cytotoxic T lymphocyte antigen 4 (CTLA-4), are more abundant in older adults than in young individuals and that may affect the crosstalk between T cells and DCs, since Tregs can prevent the maturation of DCs (9, 204–206). Age-dependent changes in T cells have, directly or indirectly, an effect on their effector functions (201). For example, the frequency of CD8^+^ T cells expressing perforin and granzyme B is reduced in older individuals, which correlated with an increased risk of severe influenza (207–209). Aged T cells can be phenotypically identified based on the expression of surface markers and intracellular molecules (e.g., transcription factors and cytokines). The co-stimulatory molecule CD28 is decreased and subsets of CD4^+^CD28^−^ and CD8^+^CD28^−^ T cells emerge in older adults (46, 210). Chronic activation of T cells also induces downregulation of CD28 expression and that has been associated with impaired vaccine responses (207, 211–214). Loss of CD27, upregulation of CD57 and KLRG-1, reduced expression of granzyme B together with telomere shortening and expression of a senescence-associated secretory phenotype (SASP), are the major hallmarks of senescent T cells (43, 46, 51, 211, 215–218). The immunosenescent T cells with a SASP phenotype play an autocrine role and promote the recruitment of pro-inflammatory innate cells that, due to aging, are not very efficient at eliminating the senescent cells. These concomitant phenomena also contribute to the establishment of inflammaging.

Dysfunction of T cells caused by T cell exhaustion has been reported in various chronic viral infections and cancers [reviewed in (219)] (75, 220–223). Chronic antigenic stimulation induces upregulation of inhibitory receptors such as programmed cell death 1 (PD-1), cytotoxic T lymphocyte antigen 4 (CTLA-4), T cell immunoglobulin and mucin domain-containing protein 3 (TIM-3), and lymphocyte activation gene 3 (LAG-3), that impair TCR signaling pathways and therefore immune T cell functions (e.g., proliferation, transcriptional signature). SAT-B1, a chromatin organizer, is downregulated in aged T cells and its expression negatively correlated with PD-1 expression in virus-specific CD8^+^ T cells (224).

Although exhaustion and senescence are two distinct phenomena that differ phenotypically and functionally, both contribute to the decline of T cell functionality during aging. Therefore, both of these processes should be considered when developing potential novel strategies to overcome dysfunctional immune responses in this age group (225).

#### B Lymphocytes

B cells undergo profound changes during aging leading to a reduced protective vaccine efficacy and reduced control of infections. They are responsible for antibody production and have effector as well as regulatory functions. Although total B cell counts remain relatively stable in the adult population, a reduced output of naive B cells from the bone marrow (BM) has been reported in mouse and human studies (226–229). Decreased production of IL-7 by stromal cells in the BM, reduces the size of the B cell progenitor population and affects B lymphopoiesis (230–232). The function of hematopoietic stem cells (HSCs) declines with age and shifts toward the generation of non-lymphoid cells, therefore reducing the source of B cell progenitors. Recently, a population of old age-associated B cells (ABC) has been identified in mice (233–237). These cells produce pro-inflammatory cytokines, in particular TNF-α, that affect the generation of mature B cells (233). In humans, accumulation of B cells with similar characteristics has been described in the peripheral blood of older individuals (238, 239). The reduced generation of naive B cells together with a progressive accumulation of dysfunctional memory B cells in the periphery, contributes to a contraction of the B cell repertoire diversity that limits the ability to recognize and respond to novel antigens.

Alterations of the immunoglobulin (Ig) class-switch recombination (CSR) and somatic hypermutation (SHM), may negatively affect the generation of high-affinity antibodies and germinal center formation, both of which are very important mechanisms for the induction of protective and long-lasting immune responses. Age-related reduction and instability of the transcription factor E47 affects the enzyme activation-induced cytidine deaminase (AID) that is involved in the regulation of CSR and SHM (62, 240). Downregulation of AID and E47, caused by the pro-inflammatory microRNAs (miR-155 and miR-16), has been observed in B cells from aging individuals (241). Reduction in size and output of the germinal centers, as a consequence of a sub-optimal T cell help to B cells, have been reported upon infection and vaccination (79, 133, 242–244). As demonstrated in older adults and aged mice, inflammation also leads to a decrease of chemokine CXCL12 production, which may impair the recruitment and accumulation of plasma cells in the bone marrow, the major site for antibody production (245, 246). The altered architecture of the spleen in older age negatively influences the humoral responses. For example, marginal zone of old mouse spleens showed a dysfunctional antigen recognition and migration of B cells due to an impaired functionality of splenic stromal cells (247). Decrease of IgM and IgD serum levels, probably due to the shift to a more differentiated and effector B cell population has been observed in humans (248–250). Finally, it has been reported that senescent B cells can produce auto-antibodies, which may lead to the development of autoimmune diseases (229, 251–253).

## Role of Viral Infections in Older Adults

Immunosenescence accounts for an increased susceptibility to viral infections such as those caused by influenza viruses and RSV and reduced vaccine efficacy in this age group. In addition, chronic stimulation of the immune system operated by viruses establishing latent infections, that can re-activate from time to time, may further impair the overall immune status in older individuals.

### Human Cytomegalovirus

Human cytomegalovirus (HCMV) is a β-herpes virus that causes lifelong latent infections in a large proportion of the human population. The mechanisms involved in HCMV latency are still poorly understood and despite extensive literature on how HCMV infection influences the adaptive immune response in older adults, this is still a matter of debate (254). Immunologically, HCMV infection is characterized by inflation of HCMV-specific memory T cells, mostly CD8^+^, with proliferation of oligoclonal T cells (96, 255–262). The accumulation of these terminally differentiated apoptosis-resistant CMV-specific T cells clones is believed to compromise the overall T cell repertoire diversity. Chronic HCMV infection also triggers an increased secretion of pro-inflammatory cytokines that favors cell damage and contributing to inflammaging, although its impact in older adults is still unclear (263–266). CMV-seropositivity has been associated with reduced immune response to (influenza) vaccination in older adults, but also in younger subjects (267, 268). Increased cardiovascular problems and mortality rate have been also reported in CMV^+^ older individuals. However, data are often inconclusive and associations are still controversial, not confirmed and do not need to have a causal relationship (267, 269–274). In contrast, CMV positivity may exert a beneficial effect by improving CD8^+^ T cell polyfunctionality, as demonstrated in healthy young individuals (263, 275, 276). Thus, chronic HCMV infections may have an impact on immune responses induced in older people after infection or vaccination. More studies are warranted to further investigate this association, which may guide the development of effective therapeutic strategies.

### Varicella Zoster Virus

Varicella zoster virus (VZV) belongs to the α-herpesvirus family (277). This virus is highly infectious (about 90% of adults are infected) and produces “varicella”, also known as “chickenpox”, a self-limiting disease which is commonly experienced during childhood and is characterized by widespread vesicular rash and fever, that usually resolves in 1-2 weeks (278–280). Despite a robust primary response, VZV is not eliminated from the body and can remain in the spinal and cranial sensory ganglia in a life-long latent state (84, 281). The mechanisms leading to latency and subsequent reactivation are still to be unraveled. However, a recent study has suggested VLT-ORF63 transcripts from VZV as potential inducers of reactivation (282). In addition, cell-mediated immunity appears to play an important role in the immune response to VZV and also latency (283–285). Reactivation occurs in 10-20% of seropositive individuals and causes the so-called “herpes zoster” (HZ) or “shingles”, a painful rash that mainly affects older adults and immunocompromised individuals, in which VZV-specific immune control has declined below a critical level (286). Even if VZV primary infection could lead to complications (e.g., bacterial infections, encephalitis, hemorrhage and pneumonia), a later reactivation process may trigger severe neurological complications, including post-herpetic neuralgia (PHN) (84, 286–289). PHN can last for years, or become permanent, with age being the most important risk factor correlating with its development. Importantly, the severity of HZ has been associated with a reduced frequency of VZV-specific effector memory T cells, highlighting the crucial role of cell-mediated immunity (290).

Three vaccines are currently licensed for preventing VZV associated disease. The most widely used VZV vaccine is a live attenuated vaccine based on the OKA-strain that can be administered in one- or two-dose regimens during childhood (286, 291–293). Although this vaccine is very effective (up to 98%) in protecting from severe VZV infection, it does not prevent latency and reactivation of the virus. Moreover, it is not suitable for immunocompromised individuals, due to a less favorable safety profile (294).

Two vaccines, Zostavax™ and Shingrix™, have been developed to prevent HZ (shingles). Zostavax™ is a live attenuated vaccine licensed in 2006 for individuals >50 years of age and contains 14-fold more of the OKA-strain than needed to prevent varicella. Several studies have indicated that the efficacy afforded by this vaccine wanes with age and appears to be inferior to that obtained with Shingrix™ (295–297). For this reason, the Zostavax™ is, effective July 2020, no longer available for use in the US. A new Zostavax™-based vaccine inactivated by gamma irradiation is under clinical investigation. Data obtained during the phase III clinical trial indicated that the vaccine is well tolerated with no significant safety issues. However, the immune response is not as strong as those induced with other vaccines and it should also be administered in a four-dose regimen (298–300).

Shingrix™ is a recombinant adjuvanted vaccine licensed in 2017 for people >50 years of age. The vaccine contains the recombinant glycoprotein E (gE) formulated with the AS01B adjuvant system. The gE is involved in viral replication, cell to cell virus transfer and is highly expressed in VZV infected cells, being also the primary target of the T cell response (279, 301–303). This vaccine is able to generate strong humoral and cell-mediated immunity, therefore overcoming the decline of the VZV-specific response observed in older individuals. The efficacy of this vaccine for preventing HZ and possible complications, such as PHN, is high irrespective of age and, importantly, the vaccine-induced immunity is long lasting and can persist up to 9 years (25, 304–306). When compared to the live-attenuated vaccine, Shingrix™ induced higher frequency of gE-specific CD4^+^ and CD8^+^ memory T cells in older adults (307). VZV-specific CD4^+^ T cells have been associated with a positive vaccine outcome (308). Shingrix™, unlike Zostavax™, has shown a favorable safety profile in immunocompromised and transplant recipients (25, 305, 306, 309, 310). However, Shingrix™ reactogenicity, due to the adjuvant, appeared to be higher compared to other vaccines used for older individuals, although serious adverse events and immune-mediated diseases were not increased in the vaccinees. The success of this vaccine for older adults may reside in the use of an adjuvant that provides a proper activation of the innate immune system, which is pivotal for the induction of an effective and long-lasting adaptive immune response.

### SARS CoV-2

Since the first reported case of COVID-19 in December 2019, the severe acute respiratory syndrome coronavirus 2 (SARS CoV-2) has infected more than 130 million people and caused over 3 million deaths worldwide (https://covid19.who.int/). SARS CoV-2 is a positive sense single stranded RNA virus that uses its S protein to bind and infect respiratory epithelial cells that express its receptor angiotensin converting enzyme 2 (ACE-2), including type II pneumocytes in the lung (311). Although the majority of infected people only develop mild disease, some develop severe disease often with a fatal outcome. Especially older people have been affected disproportionally with high case fatality rates. In the US, for example, 80% of all fatal cases were older than 65 years of age (CDC - https://www.cdc.gov/coronavirus/2019-ncov/need-extra-precautions/older-adults.html). Furthermore, the more severe course of infection observed in older people often requires hospitalization and treatment in intensive care units (312–314). Studies to elucidate the molecular and cellular mechanisms responsible for the worse outcome of SARS CoV-2 infection in aged people are ongoing. However, the strong association between age and severity of infection seems to be a common feature to other coronavirus infections (e.g., SARS CoV-1 and MERS) and it has also been observed for other respiratory viral pathogens, such as influenza viruses (315–318). Underlying co-morbidities associated with aging (e.g., cancer, hypertension, cardiovascular diseases diabetes or autoimmunity), genetic factors and reduced ability to mount adequate adaptive immune responses due to presence of dysfunctional aged immune cells, may account for the severe clinical outcome (319, 320). Pre-existing SARS CoV-2 cross-reactive T cells induced by previous exposures to seasonal human common cold coronavirus are readily detected in younger individuals, but are virtually absent in older people, suggesting that this age group may not benefit from a potential protective effect of these pre-existing cross-reactive T cells (19). A chronic, low-grade inflammation (inflammaging), the main hallmark of aging, has been suggested to play a critical role in promoting “cytokine storm” and consequent acute respiratory distress syndrome (ARDS), often observed in older individuals (321–325). The downregulation of the ACE-2 receptor together with a dysregulated angiotensin-II pathways (renin-angiotensin-aldosterone system) may foster the uncontrolled and exaggerated inflammatory response leading to pulmonary damages, multi-organ dysfunction and ultimately, death (326, 327).

The ongoing COVID-19 vaccination campaign in older subjects has already been shown very successful and significantly reduced hospitalizations and mortality among people of this age group. It also demonstrates that despite high age and associated immunosenescence, successful vaccination is still possible in this age group.

### Influenza Virus

Seasonal influenza viruses are among the leading causes of respiratory infections and responsible for 290,000-650,000 deaths annually worldwide (100). Despite the high vaccine coverage among individuals >65 years of age in some countries, this age group accounts for the vast majority of deaths and hospitalizations (CDC - https://www.cdc.gov/flu/index.htm). Although the mechanisms underlying increased severity of influenza virus infection in older individuals are still not fully understood, co-morbidities such chronic and metabolic diseases, obesity, immunosuppression, and frailty represent a risk for disease severity and development of complications (328–330).

Age-related immunosenescence affects the functionality of B and T cells, hindering an optimal protective response upon seasonal influenza vaccination. Current influenza vaccines mainly provide protection through the induction of antibodies that antigenically match the epidemic strains (331, 332). Several studies have indicated that influenza-specific antibodies in older adults decline faster, failing to maintain long-lasting protective levels (333–336). Moreover, these individuals exhibit a reduced accumulation of “*de novo*” mutations in the Ig variable gene affecting the adaptability of their antibody responses to influenza virus (337, 338). Therefore, this age group mainly rely on cross-reactive memory B cells generated early in life (339). Interestingly, a recent study has shown that influenza virus infection predominantly recalls pre-existing memory B cells against non-neutralizing epitopes in contrast to vaccination that mainly targets memory B cells to protective HA epitopes. One could speculate that boosting pre-existing immunity may play a key role in susceptibility versus protection upon influenza virus infection (339, 340). Additional defects influencing the B cell responses to influenza virus infection or vaccination are reviewed elsewhere (99).

T cells, especially CD8^+^, are an important correlate of protection against influenza virus infections also providing heterosubtypic immunity (341–344). In older adults, phenotypic and functional defects in the virus-specific CD8^+^ responses upon influenza virus infection have been demonstrated (214, 345, 346). In older adults the number of IL-7Rα^low^ effector memory CD8^+^ T cells, a subset accumulating during aging, correlated with the vaccine-induced immune response determined by antibody production. This suggests a possible implication of the IL-7Rα^low^ effector memory CD8^+^ T cell population in the aging related alterations (346).

Several studies have indicated that the functionality of the CD4^+^ T cells becomes compromised, including T follicular helper cells, which are essential for an optimal B cell response. Consequently, these functional defects impair the humoral response induced by vaccination and the formation of B cell memory (347–349). It was shown that repeated vaccinations may lead to a reduced CD4^+^ T cell response, which correlates with a reduced antibody response (350).

Strategies to increase vaccine efficacy in older adults have been developed and include the use of a high dose antigen or adjuvant and alternative administration routes and will be discussed in the next section. These improved vaccines boost not only the humoral but also cell-mediated immune responses, in contrast to the standard vaccine (351). However, the mechanisms underlying the success or failure of a vaccination strategy is still not completely understood. It will also be important to gain more insights into new correlates of protection, other than virus neutralizing antibodies, that may be more suitable for predicting influenza vaccine outcome in older adults and would guide the design of new generation influenza vaccines (208).

### Respiratory Syncytial Virus

Respiratory syncytial virus (RSV) belongs to the family of Pneumoviridae and is the leading cause of lower respiratory tract infection in infants and young children. RSV was not recognized as a potential problem in the adult population until outbreaks occurred in long-term care nursing facilities (352, 353). RSV causes significant burden of severe disease in older adults with 2-5% mortality rate (354). A relatively short-lived immunity after natural infection together with suboptimal/dysfunctional response of the “aged” immune system might be at the basis of the poor outcome of infections later in life. Despite the large number of candidate antivirals and vaccines against RSV in the pipeline, only a single antiviral treatment is approved, albeit with limited effectiveness and no vaccine has been licensed yet (355, 356). The correlates of immunological protection remain poorly understood which represents a major obstacle in RSV vaccine development, but antibodies to the F and G envelope proteins contribute to protective immunity (357–360). Both systemic and mucosal humoral immunity have been associated with protection (361–363). The frequency of RSV-specific CD4^+^ and CD8^+^ T cells is reduced in older adults compared to that in young adults (364–366). Furthermore, severity of the disease has been shown to correlate with reduced RSV neutralizing antibody titers and low numbers of CD8^+^ memory T cells (367, 368).

## Therapeutic Approaches to Increase Immunity in Older Adults

The immune system is subject to several changes throughout life, and protects against infectious pathogens, but ultimately lose functionality by e.g., immunosenescence. Immunosenescence is partly influenced by external factors such as diet and infections, but also has a genetic component (369–375). Centenarians, for instance, seem to have gene variants that allow an optimized balance between pro- and anti-inflammatory molecules (376). The genetic study of this variation could reveal information about the immunosenescence process and how it is triggered, opening a window of opportunity for potential new therapeutic treatments (53). In addition, a personalized genetic analysis could help identify which treatment will be more effective at overcoming immunosenescence on an individual basis. These aging-related hallmarks are independent phenomena but causally connected.

**Figure 2:**
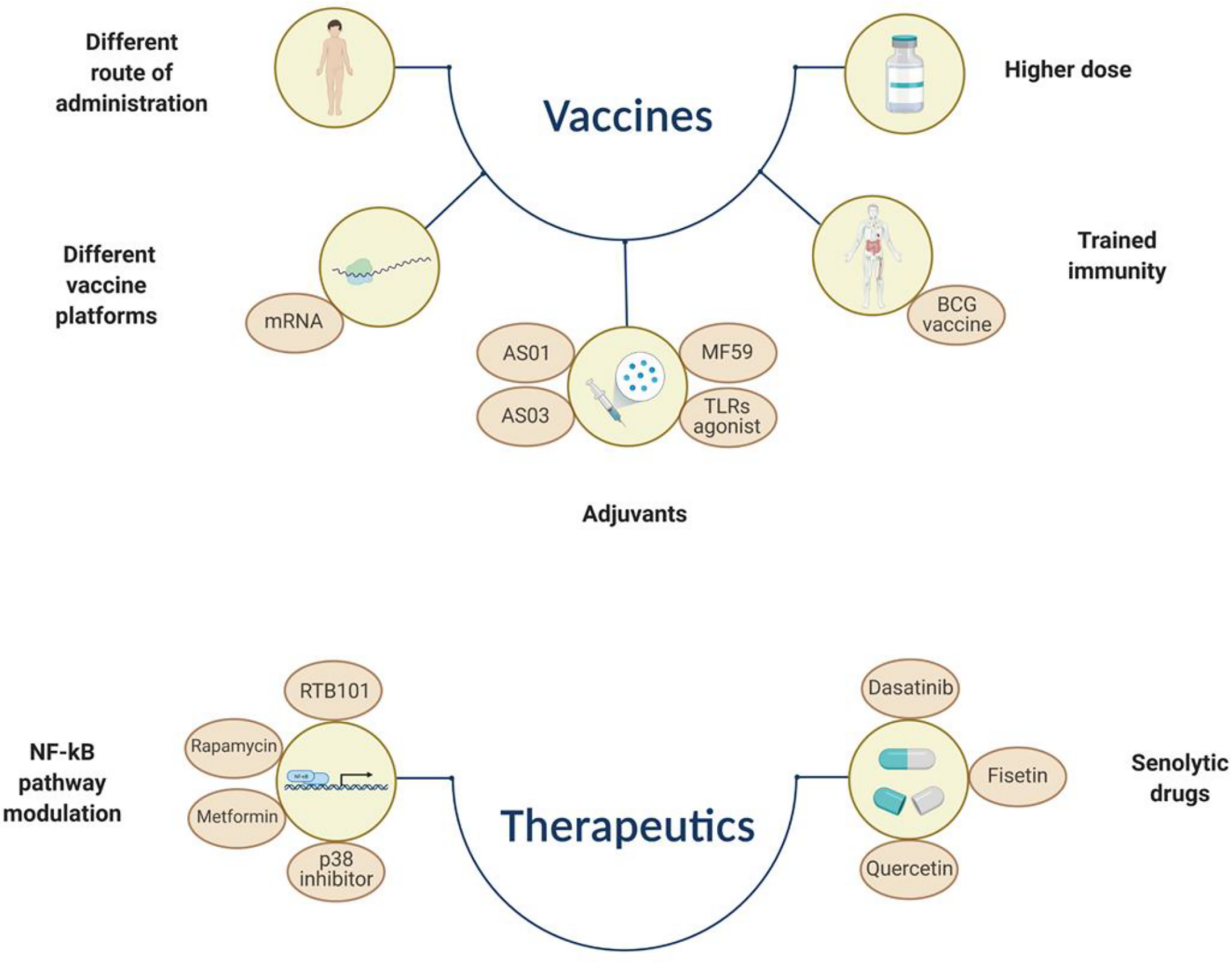
Simplified representation of different approaches that aim to overcome immunosenescence by increasing vaccine immunogenicity/efficacy or by using therapeutics that can dampen the SASP effect or induce selective apoptosis of immunosenescent cells. BCG, Bacillus Calmette–Guérin; TLR, toll like receptor. The figure was created with BioRender.com.

Several studies have provided important insights into the biological mechanisms underlying the causes of immunosenescence and have identified possible therapeutic targets (**Figure 2**) (377–379).

### Senolytic Drugs

Senescent cells are induced by cellular stress, which can occur at any age. Therefore, they can be found at any stage of life and play a beneficial role in embryogenesis, tissue repair/remodeling and tumor suppression in adulthood (380–382). Yet, secretion of soluble mediators, such as chemokines, favors the removal of senescent cells by other immune cells (e.g., macrophages and NKs) (383, 384). This process becomes less efficient with aging and results in accumulation of senescent, apoptosis-resistant cells that, together with a pro-inflammatory phenotype, contribute to tissue dysfunction and pathological manifestations (385). Chemotherapy/radiotherapy, atherosclerosis, organ transplantations or smoking, are factors among others, that accelerate immunosenescence, mainly by inducing DNA and telomere damage (386–389). In this regard, stimulation of cell death by using pharmacologically active small molecules, also known as senolytic drugs, has been identified as a promising anti-aging treatment (390). Some of these drugs inhibit the pro-survival pathways, involving the BCL-2 family members, p53 and PI3K/AKT pathways, leading to selective apoptosis of senescent cells (225, 391, 392). Dasatinib, a kinase inhibitor, and quercetin, a flavonoid targeting the PI3K/AKT, have been proven effective in eliminating senescent cells both *in vivo* and *in vitro* (393). Fisetin, another natural molecule, also appears to have senolytic properties (394). In addition, inhibitors of the chaperon HSP90, downregulate the anti-apoptotic PI3K/AKT pathway whereas a peptide altering the interaction between the transcription factor FOXO and p53, induces apoptosis of senescent cells (391, 395–398).

### NF-κB Regulators

The SASP is heterogeneous, depending on the senescent cell type or mechanism that have induced senescence. Yet, some SASP-associated cytokines and growth factors are commonly secreted by different senescent cells (e.g., IL-6, IL-8, TGF-β or activin A) (71, 399–402). Interestingly, the SASP has also been shown to include secretion of extracellular vesicles, which seem to be involved in intercellular communication and inflammatory exacerbation (403–405). In this regard, another promising therapeutic approach for targeting cellular senescence is the modulation of the NF-κB regulators, such as p53 and p38 MAPK pathways and mTOR (mammalian target of rapamycin), that can mitigate the detrimental effect of the SASP by dampening the production of pro-inflammatory cytokines (e.g., IL-1, IL-6 and IL-8) (406–408). For example, the p38 MAPK pathway has been suggested to boost immunosenescence by positively regulating IFN-γ production in T cells and by altering autophagy, which causes an increase of dysfunctional cells (409, 410). First and second generation p38 inhibitors suppress SASP expression in senescent cells (411, 412). Inhibition of the sestrin-dependent MAPK activation complex has been shown to improve T cell activity in older adults, which could be associated with an increased vaccine efficacy (413). Caloric restriction can also dampen the production of pro-inflammatory cytokines by regulating the MAPK and NF-κB pathways (414, 415). Inhibitors targeting the mTOR complex, such as the immunosuppressant drug Rapamycin and its analogues, are effective in improving the outcome of several diseases affecting older individuals (e.g., respiratory viral infections), as wells as vaccination (e.g., influenza vaccine) (407, 416). These drugs reduce the SASP while improving the innate immune response. For instance, both Rapamycin and a second-generation mTOR inhibitor RTB101, are currently under clinical investigation as possible COVID-19 treatment for adults aged >60 years of age (NCT04584710 and NCT04409327). Metformin, a widely used treatment for type II diabetes, also has been evaluated for its inhibitory effect on the SASP and as immunomodulatory agent (417). Although these drugs might offer the unique opportunity to dampen detrimental effects of aging on the immune cells or induce apoptosis of the senescent cells, it should be pointed out that their safety profile is often a matter of concern.

### Improved Vaccines

Vaccination is the most effective measure to prevent infections and reduce the disease severity but unfortunately, their effectiveness appears to be lower in older adults.

The use of adjuvants as component of improved vaccines for use in older people has proven to be another strategy to partially overcome immunosenescence by increasing immunogenicity and durability of vaccine induced immune responses. A licensed influenza vaccine containing MF59^®^, an oil-in-water emulsion, elicited higher antibody response in older individuals compared to the standard influenza vaccine and, interestingly, a broader antibody response also directed toward heterologous vaccine strains (103, 418, 419). Although the mode of action is not completely understood, MF59^®^ is believed to activate innate immune cells and favor the germinal center reaction (420, 421). Another emulsion-based adjuvant, AS03, has been licensed and used during the 2009 H1N1 influenza pandemic (422). The adjuvant system AS01, component of the herpes zoster vaccine, induces antibody and cytotoxic CD8^+^ T cell responses and also activates innate immune cells (423, 424). Several other adjuvants, including Toll-like receptors agonists, are currently in the pre-clinical phase or undergoing clinical testing and have been reviewed elsewhere (425). Additional strategies, including the use of higher dose of vaccine antigen or alternative routes of vaccine administration (e.g., intradermal instead of intramuscular), have been successfully developed for influenza vaccines targeting the older population (426, 427). The improved humoral and cell-mediated immune response achieved with such vaccines has been recently confirmed by a meta-analyses conducted on 39 clinical trials, and in a head-to-head comparison clinical trial (351, 428). Promising data have been reported in subjects of >60 years of age receiving a candidate vaccine containing a recombinant HA nanoparticle produced in insect cells and formulated with a saponin-based Matrix-M adjuvant. This vaccine was well-tolerated and induced a potent immune responses when compared to the standard high-dose inactivated influenza vaccine (429).

A messenger RNA (mRNA) vaccine against SARS-CoV-2 has been recently licensed and, interestingly, the immune responses induced by this vaccine in older adults, who are most at risk for developing severe disease, was similar to that obtained in younger people with over 90% protective efficacy in adults of >65 years old. Several factors may account for the excellent efficacy including an adequate involvement and stimulation of the innate immune system and increased vaccine uptake due to its formulation (430). More studies are warranted to unravel the immunological mechanisms governing this outstanding protective efficacy. These findings may be important for the future use of this vaccine platform to protect old individuals from other viral and maybe also bacterial pathogens.

The data obtained with the “improved” and also with the mRNA vaccines suggest that reduced vaccine efficacy might be just a limitation of currently used vaccination strategies and that intrinsic features of aging can be overcome with better vaccines. A more detailed understanding of changes affecting the immune system over time will provide fundamental insights into the biology of immunosenescence and would greatly facilitate the rational design of tailor-made vaccines for older adults. Moreover, the presence of chronic diseases and frailty, defined as a decline of function across multiple organ systems, may be better predictors of poor vaccine immunogenicity than age alone (431).

“Trained immunity” or “innate immune memory” is a recent and interesting concept that defines the ability of innate cells to form immune memory and therefore displaying characteristics of adaptive immunity (64, 432, 433). Epigenetic and metabolic mechanisms are thought to be responsible for this phenomenon. An increasing number of studies have shown that several vaccines such as those against smallpox, oral polio, measles and tuberculosis (Bacillus Calmette-Guérin, BCG), induce non-specific protective effects also against heterologous, “off-target”, infectious diseases (434, 435). Interestingly, BCG vaccination reduces respiratory infections in older adults and recent data have demonstrated that the innate immune function is less affected by aging than adaptive immunity, suggesting that trained immunity may be functional in this age group (436, 437). Moreover, BCG vaccination has been shown to increase the immunogenicity of subsequent influenza vaccination, highlighting its adjuvant-like property (438). In a recent review, “trained immunity” was proposed as a new target to enhance immune responses in older adults (439). Although data are still scarce and more insights are needed, it may represent a valuable target also for designing better vaccines. However, it cannot be excluded that the excess of cytokines induced by activation of innate cells might have negative effects on the immune system of older adults.

## Conclusions

The ongoing COVID-19 pandemic and its tremendous impact on older individuals highlights the need for the development of therapeutic and preventive measures to protect this vulnerable age group. Therefore, it is of utmost importance to advance our understanding of the mechanisms (molecular, cellular, genetic, environmental, etc.) responsible for aging and the development of immunosenescence, which will aid developing novel medical interventions. Intrinsic and extrinsic cell stress factors induce cellular senescence, which is mainly characterized by cell cycle arrest (G_0_/G_1_) and secretion of soluble factors (SASP) in the extracellular environment (440). Despite recent technological advances, phenotypical characterization of senescent cells is still challenging due to a lack of defined and univocal/universal biomarkers but also for their heterogeneous nature and dynamic evolution. It is still not clear how many different subsets of senescent cells can be identified and their distribution in tissues and periphery. Although some interesting animal models to study cellular senescence have been developed, their results might not be easily translated to the human situation (441, 442). To obtain a better insight into the complexity of immune ageing/immunosenescence and thus the possibility to decipher and dissect mechanisms and cells involved, requires the use of multiple scientific approaches and disciplines (e.g., high-resolution omics-technologies, multiparametric analyses, system biology and big data analyses). With the information obtained, better prophylactic and therapeutic interventions can be developed to prevent deterioration of the health status, often associated with aging and immunosenescence. This way, the quality of life in this age group can be maintained and mortality, including that caused by infectious agents, reduced.

## Author Contributions

MP-P, AO, TB, HE, GR, and GS conceptualized and composed the manuscript. GR and GS supervised all aspects of the manuscript preparation. All authors contributed to the article and approved the submitted version.

## Funding

This work was supported by the Alexander von Humboldt Foundation in the framework of the Alexander von Humboldt Professorship endowed by the German Federal Ministry of Education and Research; the European Union’s Horizon 2020 research and innovation program under grant agreement No 848166 (ISOLDA) and the German Research Foundation, Excellence Strategy program–EXC 2155 Resolving Infection Susceptibility, “RESIST”.

## Conflict of Interest

The authors declare that the research was conducted in the absence of any commercial or financial relationships that could be construed as a potential conflict of interest.
